# Best Evidence Summary of Folic Acid Supplementation for Prevention of Neural Tube Defects in Women of Childbearing Age

**DOI:** 10.3390/nu18040641

**Published:** 2026-02-15

**Authors:** Jiahe Li, Bihui Chen, Ning Liu, Wenjia Dong, Dandan Lv, Shuangjin Li, Xiu Zhu

**Affiliations:** 1 School of Nursing, Peking University, Beijing 100191, China; ljh20000807@126.com (J.L.); 2310108126@stu.pku.edu.cn (B.C.); liuning@bjmu.edu.cn (N.L.); 2411210149@stu.pku.edu.cn (D.L.); 2411220024@stu.pku.edu.cn (S.L.); 2 PLA Rocket Force Characteristic Medical Center, Beijing 100088, China; 13718753941@163.com

**Keywords:** folic acid supplementation, neural tube defects, periconceptional care, evidence-based medicine, best evidence

## Abstract

Objectives: To summarize the best evidence regarding folic acid supplementation for preventing neural tube defects (NTDs) in women of childbearing age and to develop a structured evidence summary for guiding clinical practice. Methods: We systematically searched multiple databases and professional websites from 1 January 2013 to 18 September 2025. Sources included 7 databases and 20 professional websites. The search targeted clinical guidelines, expert consensuses, best practices, and recommended practices on folic acid supplementation for NTD prevention in women of childbearing age. The retrieved literature underwent quality assessment, evidence extraction, and summarization. Results: The review included 17 publications: 10 guidelines, 4 expert consensuses, 2 recommended practices, and 1 best practice. From these, 14 distinct evidence statements were synthesized and organized into five thematic dimensions: risks of neural tube defects and the role of folic acid, time window of neural tube closure, timing and dosage of folic acid supplementation, relationship between dietary folic acid and folic acid tablets, and folic acid-related testing. The key recommendations include initiating supplementation at least 3 months preconception, with daily doses of 0.4 mg for low-risk, 1.0 mg for moderate-risk, and 4.0–5.0 mg for high-risk women, continuing through the first trimester, emphasizing that dietary intake alone is insufficient, and advising against routine folate testing. Conclusions: This study synthesized the best available evidence regarding folic acid supplementation for preventing NTDs in women of childbearing age, providing an evidence-based foundation to inform clinical practice, particularly for healthcare systems and populations in regions without mandatory folic acid food fortification.

## 1. Introduction

Neural tube defects (NTDs) constitute a major global public health issue, with a prevalence at birth of 18.6 per 10,000 live births worldwide. Approximately 260,100 new cases occur annually, and 75% of individuals born with an NTD die before reaching the age of five [1]. Beyond the profound impact on individual health, NTDs impose substantial economic and psychological burdens on families and place a heavy strain on healthcare system for long-term treatment and management [2]. The prevalence of NTDs demonstrates significant geographical and socioeconomic variation, with low- and middle-income countries bearing a disproportionately heavy burden [3]. NTDs, such as anencephaly and spina bifida, arise from the failure of the embryonic neural tube to close normally, a process typically concluded by the 28th day post-conception. These defects can lead to fetal death, stillbirth, or severe lifelong disability. Notably, folic acid (vitamin B9), an essential nutrient that must be obtained from the diet or supplements, is pivotal in preventing these defects when taken adequately during the periconceptional period, underscoring its role as a primary prevention strategy [4].

However, a significant gap exists globally between folic acid supplementation practices and scientific evidence. A meta-analysis revealed that preconception supplementation rates are suboptimal, remaining below 50% in developed countries and consistently falling below 25% in low- and middle-income countries [5]. A study in China found that only 16.1% of pregnant women took folic acid at the correct time [6]. This low compliance rate is closely linked to low awareness levels among women of childbearing age regarding folic acid. International studies confirm this widespread knowledge gap: in South Korea, only 23.7% of women clearly understood folic acid’s role in preventing NTDs and the correct timing for its supplementation [7]; while a study in Saudi Arabia found that merely 55.7% of female university students were aware of the appropriate intake timing [8]; furthermore, research across six economically underdeveloped provinces in China revealed that only 15% of women mastered core knowledge including folic acid’s preventive effect, optimal intake timing, methods, and dosage [9].

This gap in knowledge is reflected in a high prevalence of biochemical folate insufficiency. A systematic review of global data (2000–2014) found that folate deficiency (serum folate < 10 nmol/L) affected >20% of women of reproductive age in many lower-income countries, while folate insufficiency (red blood cell folate < 906 nmol/L for NTD risk reduction) exceeded 40% in most countries surveyed [10]. Specifically in China, a national assessment covering 2000–2020 reported a median serum folate deficiency rate of 15.0% and a median red blood cell folate insufficiency rate as high as 49.0% among women of childbearing age [11], with particularly low concentrations observed in northern and rural regions [12].

These findings underscore that suboptimal folate status is not merely a biochemical issue but is intrinsically linked to broader determinants of health and information access. At its root, the low awareness of folic acid knowledge among women of childbearing age stems from the combined effects of health determinants and contemporary information environment characteristics. Research consistently indicates that structural factors such as low educational attainment, low income, and limited healthcare accessibility are core barriers constraining the acquisition and understanding of folic acid supplementation knowledge [5,6,7,8]. However, in the digital age, this issue presents new characteristics: the internet has become the primary channel for women of childbearing age to obtain health information [7,8]. While these channels have broken down some geographical and economic barriers, they have introduced new challenges such as information overload, inconsistent quality, and the proliferation of misleading content. Although scientifically rigorous clinical practice guidelines exist, their comprehensive and highly specialized content—often embedding core recommendations within complex diagnostic and therapeutic contexts—makes them lengthy and impractical for public health advocacy, health education materials, and rapid consultation by primary healthcare workers.

Therefore, overcoming this challenge hinges on systematically extracting, synthesizing, and translating the core evidence scattered across high-quality guidelines. Currently, there is a lack of a comprehensive, authoritative, clear, and widely accessible evidence knowledge base and personalized support tools for folic acid supplementation [13]. Given this, there is an urgent need to synthesize the best available evidence on folic acid for preventing fetal NTDs, establish a standardized knowledge base, and develop new health education approaches to effectively enhance their folic acid-related knowledge and ultimately improve intake behaviors.

This study aimed to systematically retrieve, screen, and evaluate literature, then extract and synthesize evidence, thereby developing a structured, easily accessible and applicable knowledge base for guiding folate acid intake among women of childbearing age. This knowledge base will provide direct, reliable core knowledge support for developing evidence-based health education materials and clarifying key points for clinical counseling. Ultimately, it will serve clinical practice and public health interventions, helping to bridge the gap between knowledge, belief, and action.

## 2. Materials and Methods

### 2.1. Literature Inclusion and Exclusion Criteria

Studies were included if they met the following criteria: (1) focused on folic acid supplementation for the prevention of NTDs; (2) were guidelines, expert consensuses, best practices, or recommended practices; (3) were developed using evidence-based or formal consensus methodologies (guidelines without evidence grading or recommendation strength were defined as “non-evidence-based” [14]); (4) were the latest version available; and (5) were published in Chinese or English.

Studies were excluded if they were: (1) direct translations, duplicates, or interpretations of existing guidelines; or (2) unavailable in full text.

### 2.2. Literature Search Strategy

A systematic search was performed following standard methodology for evidence synthesis. We employed a combination of subject headings and free-text terms in both English and Chinese. English terms included, but were not limited to: “folic acid,” “folate,” “preconception care,” “neural tube defects,” “anencephaly,” and “spina bifida.” Corresponding Chinese terms were used in Chinese databases.

Development of the search strategy was conducted with the assistance of an information specialist from the university library, who had expertise in systematic review search methodology. The initial strategy was drafted by two reviewers (J.L. and B.C.) who have received formal training in systematic review methodology. This draft was then refined through iterative pre-searches and discussions with the information specialist and the research team to optimize sensitivity and specificity.

The robustness of the strategy was ensured through the following steps: (1) consultation with an information specialist; (2) multiple rounds of pre-search testing and refinement; and (3) review and approval by the senior researcher (X.Z.) overseeing the project.

Comprehensive searches were conducted in 7 electronic databases (i.e., PubMed, Embase, CINAHL, Web of Science, CNKI, Wanfang, VIP) and 20 websites of major professional organizations (e.g., UpToDate, National Institute for Health and Care Excellence (NICE), SIGN (Scottish Intercollegiate Guidelines Network), ACOG (American College of Obstetricians and Gynecologists), SOGC (Society of Obstetricians and Gynaecologists of Canada)). The detailed search strategies for all sources are provided in Appendix A.

The initial search covered the period from 1 January 2013 to 31 December 2023. An updated search was performed on 18 September 2025 to identify any newly published guidelines. Although 28 new records were identified, only one recent guideline [15] was deemed relevant. Upon full-text review, its recommendations were found to be derived from evidence already captured in our initial search; consequently, no additional publications were included from the update.

### 2.3. Literature Screening and Data Extraction

Two reviewers, who had received training in Joanna Briggs Institute (JBI) evidence-based methodology and had experience in conducting systematic reviews, independently performed the study selection and data extraction. Any discrepancies were resolved through consensus or, when necessary, by consulting a third reviewer.

The selection process was managed using EndNote 20 software. After removing duplicates, the reviewers screened titles and abstracts against the inclusion criteria. The full texts of potentially relevant records were then retrieved and assessed for eligibility. A list of studies excluded after full-text review, along with the primary reasons for exclusion, is provided as Supplementary Material S1. Additionally, the reference lists of all included publications were manually searched to identify any additional relevant studies.

A standardized data extraction form was developed and used to extract key information from the included documents, including the title, publication/update year, author(s)/issuing body, source of publication, journal of publication, country/region, and recommendations related to folic acid and NTDs prevention.

### 2.4. Literature Quality Assessment

The quality of the included evidence was critically appraised using standardized tools by two independent reviewers. Any discrepancies were resolved through discussion or with input from a third researcher.

Clinical guidelines were assessed using the Appraisal of Guidelines for Research and Evaluation II (AGREE II) instrument. This tool comprises 23 items across 6 domains: scope and purpose, stakeholder involvement, rigor of development, clarity of presentation, applicability, and editorial independence. Each of the 23 items is rated on a 7-point scale, where a score of 1 indicates an absence of relevant information or very poor reporting, and a score of 7 represents high-quality reporting that fulfills all criteria. Scores between 2 and 6 reflect varying levels of reporting completeness and quality. The score for each domain was calculated as the sum of the scores of all items within that domain. This raw score was then standardized using the AGREE II standard formula: Standardized Score (%) = (Obtained Score − Minimum Possible Score)/ (Maximum Possible Score − Minimum Possible Score) × 100.

As the AGREE II user manual does not define thresholds for standardized domain scores indicating high guideline quality, this study extensively referenced multiple guideline quality assessment studies [16,17,18]. Based on standardized domain scores, we defined the following criteria for recommendation: (1) Grade A (Recommended): Standardized scores ≥ 60% in all 6 domains; (2) Grade B (Recommended with modifications): Standardized scores are between 30% and 60% in most domains (≥3); (3) Grade C (Not recommended): ≥3 domains with standardized scores < 30%. Only guidelines categorized as Grade A or B were included for evidence extraction.

To ensure consistency, inter-rater reliability was quantified using the Intraclass Correlation Coefficient (ICC). The ICC was interpreted as follows: <0.40, poor; 0.40–0.75, moderate; and >0.75, high agreement [19].

Expert consensuses, best practices, and recommended practices were appraised using the corresponding JBI critical appraisal checklist for text and opinion papers [20]. This tool comprises six items that assess the credibility and logical development of the expert opinion. In this system, a lower grade number indicates a higher level of evidence (e.g., Level 1 represents the highest level of evidence, such as evidence from systematic reviews of randomized controlled trials, while Level 5 represents the lowest, such as evidence from expert opinion or laboratory research). Two reviewers independently evaluated each document, rating the items as “Yes,” “No,” “Unclear,” or “Not Applicable.” A document was included only if it achieved a pre-established quality threshold, which required affirmative (“Yes”) responses to a majority of the items, with particular emphasis on the logical derivation of the conclusions.

### 2.5. Evidence Translation and Synthesis

Recommendations regarding folic acid intake for the prevention of NTDs were extracted from the included literature by two researchers working independently and concurrently. The extractions were then cross-checked. Any discrepancies identified were resolved through discussion between the two researchers, or by adjudication from a third researcher when consensus could not be reached. For recommendations published in English, both researchers independently translated them into Chinese. We compared these translations and discussed them to create a consensus version.

The synthesis of all recommendations (both translated and original) into the final evidence summary was guided by the following principles: (1) for identical recommendations, select the most clearly articulated version; (2) for conflicting recommendations, trace the evidence sources and prioritize the one based on the most recent and highest-quality evidence; (3) for complementary recommendations, merge their content into a comprehensive statement; and (4) for independent recommendations, retain the original statement. This methodology was adapted from established practices for evidence synthesis [21]. Throughout this synthesis, true conflicts between recommendations were rare. When they occurred, they were resolved by examining the publication date, the hierarchy of evidence underpinning the recommendation, and the consistency with broader public health principles. The selection of the most clearly articulated version from among substantively identical recommendations was based on pre-defined criteria of completeness, precision, and clinical applicability.

## 3. Results

### 3.1. Literature Search Results and Overview

**Figure 1:**
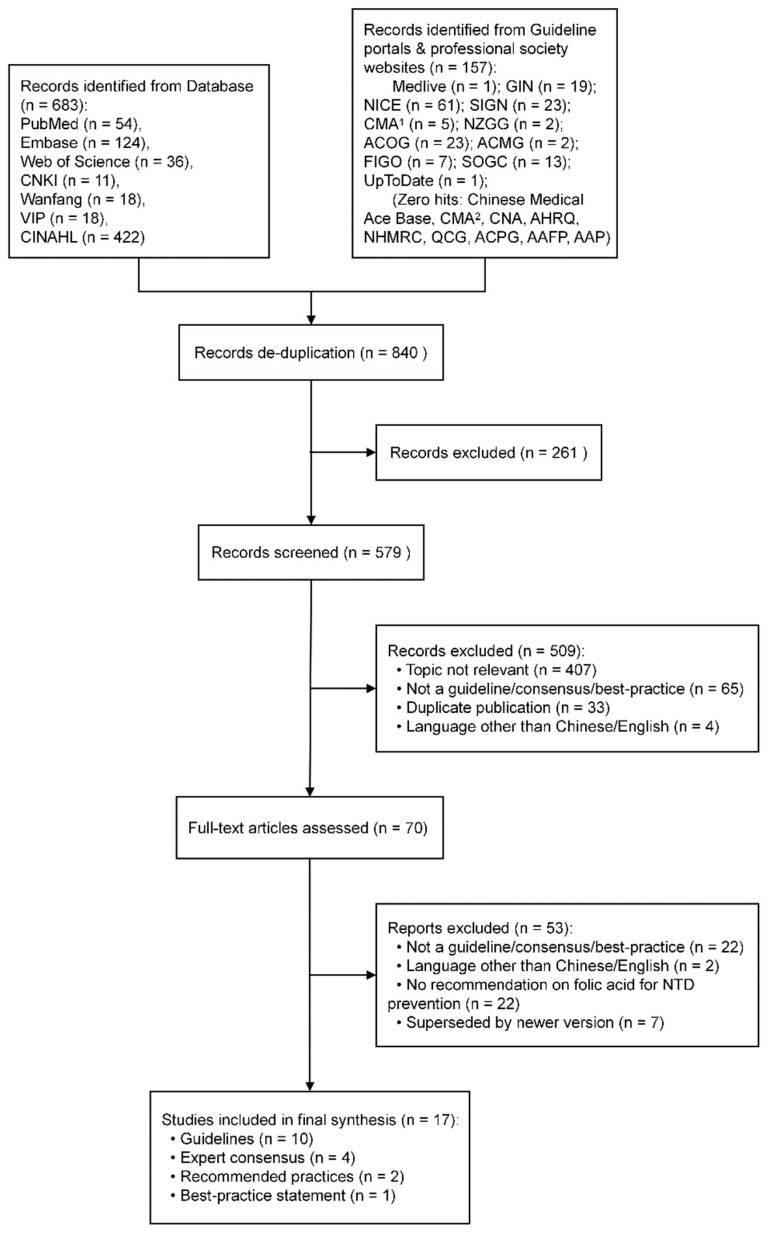
The literature searching flowchart. Notes: CNKI (China National Knowledge Infrastructure); Wanfang (Wanfang Data); VIP (Chongqing VIP Information Co., Ltd.); CINAHL (Cumulative Index to Nursing and Allied Health Literature); GIN (Guidelines International Network); NICE (National Institute for Health and Care Excellence); SIGN (Scottish Intercollegiate Guidelines Network); CMA^1^ (Canadian Medical Association); NZGG (New Zealand Guidelines Group); ACMG (American College of Medical Genetics and Genomics); ACOG (American College of Obstetricians and Gynecologists); FIGO (International Federation of Gynecology and Obstetrics); SOGC (Society of Obstetricians and Gynaecologists of Canada); CMA^2^ (Chinese Medical Association); CNA (Chinese Nursing Association); AHRQ (Agency for Healthcare Research and Quality); NHMRC (National Health and Medical Research Council); QCG (Queensland Clinical Guidelines); ACPG (Australian Clinical Practice Guidelines); AAFP (American Academy of Family Physicians); AAP (American Academy of Pediatrics).

The systematic search identified 840 records. After the removal of 261 duplicates, 509 records were excluded following title and abstract screening. A further 53 publications were excluded after full-text assessment, resulting in a final inclusion of 17 publications for evidence synthesis. The included literature comprised 10 clinical practice guidelines, 4 expert consensus statements, 2 recommended practice documents, and 1 best practice document. The study selection process is detailed in the PRISMA flow diagram (Figure 1), and the basic characteristics of the included publications are summarized in Table 1.

### 3.2. Included Guideline Quality Assessment Results

Among the included guidelines, three guidelines were rated as Grade A (recommended), five as Grade B (recommended with modifications), and two as Grade C (not recommended) which were excluded. The inter-rater reliability for the AGREE II assessments was high, with ICC values ranging from 0.773 to 0.908 across all evaluated guidelines, all exceeding the threshold of 0.75 for high agreement. The detailed scores for each domain are presented in Table 2.

### 3.3. Inclusion of Expert Consensus, Best Practice, and Recommended Practice Evaluation Results

All four expert consensus documents, two recommended practice documents, and one best practice document met the pre-specified quality threshold in the JBI critical appraisal and were therefore included for evidence extraction. The results of the quality evaluation for these documents are presented in Table 3.

### 3.4. Best Evidence Summary

By systematically synthesizing evidence regarding folic acid intake for the prevention of NTDs in women of childbearing age, we integrated findings across five key thematic dimensions: (1) the harm of NTDs and the role of folic acid; (2) the time window of neural tube closure; (3) the timing and dosage of folic acid supplementation; (4) the relationship between dietary folate and folic acid supplements; and (5) folic acid-related testing. This synthesis yielded 14 distinct best practice evidence statements, which were graded using the JBI evidence pre-classification and evidence rank system (2014) [39] (see Section 2). To facilitate the interpretation of the evidence on supplementation dosage, the risk categories for NTDs used in this review are defined in Table 4. The evidence statements regarding folic acid dosage, stratified by these risk categories, are presented in Table 5.

## 4. Discussion

### 4.1. Rigor and Scientific Soundness of the Evidence Summary Process

The development of this evidence summary adhered to a rigorous and systematic methodology to ensure its scientific soundness. The work was conducted by a team comprising two master’s degree candidates and one specialist in nursing education and research, all of whom have undergone formal training in evidence-based nursing and evidence translation methodology and have declared no conflicts of interest.

The process involved a comprehensive review of the literature on folic acid supplementation for preventing NTDs in women of childbearing age. Two researchers independently executed the systematic search, screening, quality appraisal, and data extraction from guidelines, expert consensuses, best practices, and recommended practices. The final evidence statements were derived through consensus meetings. The quality appraisal results affirm the reliability of the included evidence. Of the ten identified guidelines, seven met the quality threshold for inclusion: three [24,25,28] were rated as Level A (recommended) and five [22,23,26,27,29] as Level B (recommended with modifications), indicating that they were developed using rigorous and reliable methodologies. The two guidelines rated Level C (not recommended) [30,31] were excluded. Furthermore, all four [33,34,35,36] expert consensuses, one best practice [32], and two recommended practices [37,38] were assessed to be of high credibility and were retained.

This process culminated in a summary of 14 best evidence statements, organized across 5 domains. The entire process—from retrieval and screening to evaluation and extraction—was conducted according to strict principles of transparency and scientific rigor, under the supervision and final review of a senior nursing expert to ensure an objective and accurate synthesis of the current best evidence.

It is also noteworthy that our synthesized recommendations align with the position of major international public health bodies. For instance, the WHO also advocates for daily periconceptional folic acid supplementation (400 μg) for all women planning pregnancy to prevent NTDs [42,43]. This consensus across international and national guidelines underscores the robustness and global relevance of the evidence base supporting risk-stratified folic acid supplementation, particularly in unfortified populations.

### 4.2. Knowledge Base on Folic Acid Intake for Preventing Neural Tube Defects in Women of Childbearing Age

#### 4.2.1. The Risks of Neural Tube Defects and the Role of Folic Acid

The synthesized evidence underscores the severe clinical implications of NTDs and establishes the preventive role of folic acid. Specifically: NTDs represent a group of major congenital anomalies arising from the failure of normal neural tube closure during early embryogenesis. The primary clinical manifestations include anencephaly, spina bifida, and encephalocele. Anencephaly and severe encephalocele are conditions that most commonly lead to fetal death or stillbirth. In the rare instances where live birth occurs, affected newborns typically survive for only a very short period or present with severe neurological deficits. Children with spina bifida and mild encephalocele may survive but cannot be cured, often leading to lifelong disabilities such as lower limb paralysis, urinary and fecal incontinence, and intellectual impairment. Children with spina bifida are also prone to hydrocephalus, with many succumbing to premature death [22,37]. Folic acid cannot be synthesized in the human body and must be obtained from external sources. Foods rich in natural folate include dark green vegetables, citrus fruits, legumes, nuts, and animal liver. Folic acid added to medications, supplements, and fortified foods is typically synthetic folic acid [22]. A substantial body of evidence confirms that additional folic acid intake and consumption of folate-rich diets effectively reduce the occurrence and recurrence of NTDs [22,23,26,32,37].

#### 4.2.2. Timing of Neural Tube Closure and the Significance of Preconception Folate Intake

The evidence summary highlights a critical embryological time window that dictates the necessity of preconception supplementation. This is based on the following understanding: Normally, the human embryonic neural tube closure begins on day 21 after conception (equivalent to day 35 after the last menstrual period) and completes closure by day 28 (equivalent to day 42 after the last menstrual period). If maternal folate levels are insufficient during this period, fetal neural tube closure may fail, consequently leading to NTDs [22,23,32].

#### 4.2.3. Timing and Dosage of Folic Acid Supplementation

Overall, balanced diets, appropriate use of folic acid supplements, and food fortification are effective means to improve folate nutritional status [33]. Even when selecting folic acid supplements or fortified foods, a balanced diet should remain the foundation. Folate metabolism in the body is influenced by multiple factors. When developing a folic acid supplementation plan (including dosage, timing, type, etc.), it is necessary to consider factors such as genetics (MTHFR C677T genotype), physiological state (age, gender), disease, medication use, lifestyle (folate intake from diet, alcohol consumption), and the status of other related nutrients to achieve personalized supplementation [33].

Supplementation dosage is stratified by an individual’s risk of NTDs, as defined in Table 4. Women without high-risk factors who are planning or may become pregnant should begin daily intake of folate-rich foods and supplementation with 0.4 mg of folic acid at least three months before anticipated conception and continue through the first trimester of pregnancy. For the second and third trimesters and during lactation, the recommended folic acid supplement dose is 0.4 mg/day [22,23,25,28,29,33,34,35,36,37,38]. Given that many pregnancies are unplanned, many individuals may be unaware of their pregnancy during the critical period. To fully benefit from supplementation, daily folic acid supplementation is recommended for all women of childbearing potential.

Women in the moderate-risk group require consumption of folate-rich foods and daily oral supplementation with a multivitamin containing 1.0 mg folic acid. This regimen should be initiated at least three months prior to conception and continued until the 12th week of pregnancy. For the second and third trimesters and during lactation, the recommended supplemental folic acid dose is 0.4 mg/day [22,29,33].

High-risk groups should consume folate-rich foods and begin taking a daily supplement of 4.0 mg of folic acid (or 5.0 mg, as 4 mg formulations are unavailable in China) at least one month before conception and continue until the 12th week of pregnancy. Additionally, from 12 weeks of pregnancy onwards, continue daily supplementation with a multivitamin containing 0.4 to 1.0 mg of folic acid throughout the entire pregnancy and for 4 to 6 weeks postpartum or until breastfeeding ceases [22,23,24,29,33,35,36].

Personalized supplementation may be considered for specific circumstances, such as residence in northern China (especially rural areas), low dietary intake of fresh vegetables and fruits, low blood folate levels, the TT genotype at the DMTHFR 677 locus, or short preconception planning periods. In such cases, supplement dosage may be increased or preconception supplementation duration extended as appropriate [22,33]. For women with hyperhomocysteinemia, daily supplementation of at least 5 mg folic acid is recommended until blood homocysteine levels normalize before considering conception. This 5 mg daily supplementation should continue until the end of the third month of pregnancy. During the second and third trimesters and throughout lactation, the recommended folic acid supplementation dose is 0.4 mg/day.

Additionally, women should be advised against obtaining high-dose folic acid supplementation through multivitamin supplements, as this may lead to harmful levels of other vitamins, such as vitamin A, which is teratogenic. Prenatal vitamins taken once daily combined with 1 mg folic acid tablets taken three times daily provide a total daily folic acid intake of 4 mg. Taking three 1 mg folic acid tablets/capsules at once is more convenient [32]. Regarding folic acid safety, the tolerable upper intake level (UL) for adults is generally 1 mg. Preventive supplementation of 4 mg for women at high risk of NTD pregnancy is typically considered non-toxic in the short term. However, the dose should be reduced after early pregnancy, as it no longer serves to prevent NTDs at this stage. Furthermore, the possibility of adverse effects on the fetus from long-term high-dose exposure cannot be definitively ruled out [32].

#### 4.2.4. The Relationship Between Dietary Folate and Folic Acid Supplements

Dietary intake alone cannot meet requirements, making folic acid tablet supplements necessary. The human body cannot synthesize folate and must obtain it from food, while the increased demands during pregnancy and fetal development further elevate requirements. Although folate in natural foods is relatively safe, its structure is unstable (easily destroyed during food processing) and its bioavailability is low, reaching only about 60% of synthetic folic acid. Absorption and utilization in the body are further affected by factors such as medications, alcohol, and deficiencies in other nutrients. Even for the general population, achieving adequate folate intake presents challenges. Particularly for high-risk groups prone to folate deficiency, such as populations in northern China, impoverished rural areas, winter/spring seasons, pregnant/lactating women, individuals on long-term folate-antagonistic medications, heavy drinkers, those with certain diseases, or those with folate metabolism gene variants, additional folic acid supplementation is recommended to address deficiency/insufficiency [29,33]. Dietary folate sources include asparagus, spinach, broccoli, soybeans, citrus fruits, dried fruits, beef liver, leafy greens, legumes, avocados, eggs, dairy products, barley, tofu skin, dried bean curd sticks, walnuts, garlic sprouts, peanuts, rapeseed, fennel, red amaranth, chrysanthemum greens, chicken eggs, and duck eggs [22,32,33].

#### 4.2.5. Folate-Related Testing

Routine folate metabolism testing is generally not recommended, nor is MTHFR polymorphism testing, as daily supplementation with 0.4 mg of folic acid effectively increases folate concentrations in red blood cells regardless of test results [27,32]. Additionally, routinely monitoring folate levels is unnecessary for reproductive-age women supplementing with folate [32]. However, if folate deficiency arises not from dietary insufficiency but from known comorbidities (e.g., inflammatory bowel disease or bariatric surgery), monthly serum folate monitoring is warranted to ensure adequate supplementation (serum folate levels of 28–30 nmol/L) [32].

### 4.3. Implications of Food Fortification Policies for Evidence Interpretation

A critical consideration in interpreting the evidence synthesized in this review is the varying context of mandatory folic acid food fortification across the jurisdictions from which the included guidelines originate. Notably, six of the included guidelines are from the United States and three from Canada, countries that have implemented mandatory fortification of cereal grains since 1998 [44,45]. In contrast, guidelines from China and the United Kingdom (comprising four and three documents, respectively) were developed in contexts without nationwide mandatory fortification, although the UK has recently passed enabling legislation [46].

This policy landscape directly informs the applicability and intended audience of our evidence summary. The recommendations presented here are of paramount importance for healthcare providers, public health planners, and women of childbearing age in regions without mandatory fortification or where fortification is not reliably implemented. In such settings, daily folic acid supplementation is the principal, evidence-based strategy to achieve adequate folate status for NTD prevention. Our risk-stratified dosage guidance (0.4 mg, 1.0 mg, 4–5 mg) provides a clear clinical protocol for these populations.

For regions with established and effective fortification programs, our risk-stratified supplementation recommendations remain valid but require contextualization. Individuals consuming fortified foods will have a baseline intake of folic acid. Therefore, healthcare providers should consider total intake from both diet and supplements, particularly for women in the high-risk category prescribed 4–5 mg/day, to align with the general principle of avoiding excessively high intakes. Although the short-term use of high-dose folic acid for NTD prevention is considered safe, ongoing research discusses the biological effects of sustained high folate levels [47], underscoring the importance of dose reduction after the first trimester as recommended in our evidence statements.

Furthermore, specific subgroups, such as individuals adhering to gluten-free diets that avoid fortified wheat products, may not benefit from population-wide fortification. For them, the supplementation guidelines summarized here are directly applicable and essential.

In conclusion, while our evidence summary provides a robust set of universal recommendations based on the best available guidelines, its implementation should be nuanced by local public health policy. Future iterations of such evidence syntheses could benefit from explicitly grading or annotating recommendations based on the fortification context of the source guideline. The FFINetwork (https://ffinetwork.org/) (accessed on 30 January 2026) serves as a valuable resource for tracking global fortification policies and should be consulted by policymakers and practitioners aiming to implement these recommendations.

### 4.4. Strengths and Limitations of This Study

This study possesses several key strengths: (1) Comprehensive and systematic approach: Focusing on folic acid supplementation for preventing NTDs in women of childbearing age, this work systematically integrated clinical guidelines and expert consensus to construct a structured, actionable summary of the best available evidence. It comprehensively retrieved and synthesized guidelines, consensus statements, and recommended practices from multiple countries, including China, the United States, the United Kingdom, and Canada, ensuring broad and representative evidence sources. (2) Methodological rigor: The study employed internationally recognized quality assessment tools—AGREE II for guidelines and JBI criteria for expert consensus documents—with high inter-rater reliability (ICC > 0.75). Evidence extraction and synthesis were performed independently by two reviewers and reviewed by experts, ensuring methodological standardization and result reliability. (3) High-quality and well-structured evidence output: A total of 17 high-quality publications were included, among which 10 were high-quality clinical guidelines. The 14 evidence statements were clearly graded using the JBI evidence grading system and organized into five thematic dimensions, facilitating efficient comprehension and application by clinicians and public health practitioners. (4) Strong clinical relevance and guidance value: The evidence summary not only distinguishes supplementation regimens for low-, medium-, and high-risk populations but also explicitly recommends against routine folate metabolism testing and serum monitoring, thereby helping to avoid unnecessary medical interventions and reflecting an evidence-based, patient-centered approach.

This study also has several limitations: (1) Language restriction: Only guidelines and consensus documents published in Chinese and English were included, which may have led to the omission of relevant high-quality evidence available in other languages. (2) Exclusion of primary research and systematic reviews: To maintain focus on consolidated recommendations and consensus, original studies and systematic reviews were not incorporated. While this strengthens the authority of the recommendations, it may not reflect the most recent research advances or region-specific evidence not yet captured in formal guidelines. (3) Subjective interpretation in quality appraisal: Although reviewers were trained and cross-checked, the evaluation of expert consensus documents relied on researchers’ interpretation of JBI criteria, which may introduce a degree of subjectivity. (4) Lack of implementation context analysis: The study primarily focused on synthesizing the “best evidence” content and did not systematically examine socioeconomic, cultural, or behavioral barriers that may affect adherence to folic acid supplementation. Future work should integrate implementation science approaches to better support the translation of evidence into practice. (5) Initial lack of contextual analysis for food fortification policies: Our study design did not systematically analyze the impact of mandatory food fortification policies on the included guidelines. Future evidence syntheses on public health interventions should incorporate such contextual analysis from the outset.

## 5. Conclusions

Through a systematic synthesis of evidence, this study has developed a comprehensive knowledge base on folic acid intake for preventing NTDs in women of childbearing age, providing a scientific foundation for clinical practice. A total of 17 high-quality documents were identified, including 10 clinical guidelines, 4 expert consensuses, 2 recommended practices and 1 best practice. The synthesis results in 14 key evidence statements, organized across five critical dimensions: the risks of NTDs and the role of folic acid; the time window of neural tube closure; the timing and dosage of folic acid supplementation; the relationship between dietary folate and folic acid supplements; and folate-related testing.

For clinical practice, the evidence strongly supports a risk-stratified supplementation strategy: (1) all women of childbearing age should take 0.4 mg of folic acid daily starting at least 3 months before conception; (2) women at moderate risk require 1.0 mg daily; and (3) high-risk women require 4–5 mg daily. The findings also affirm that dietary sources alone are insufficient to meet recommended levels for NTD prevention, and routine serum or metabolic testing for folate is not recommended for most women of childbearing age. These consolidated insights provide a robust, evidence-based resource to guide future educational initiatives and research on folic acid supplementation. This guidance is especially critical in regions without mandatory folic acid food fortification, where supplementation serves as the primary preventive strategy. Implementation of these recommendations should be informed by local fortification policies to tailor counseling and prevent potential over-supplementation in settings where foods are already fortified.

## Figures and Tables

**Table 1:** Basic characteristics of the included literature (*n* = 17).

No.	Literature Title	Publication/Update Year	Author/Publishing Organization	Literature Type	City, Country
1	Guideline for the Prevention of Neural Tube Defects by Periconceptional Folic Acid Supplementation (2017) [22]	2017	Periconceptional Folic Acid Supplementation to Prevent NTDs Guideline Working Group	Guideline	Beijing, China
2	Neural Tube Defects [23]	2017	ACOG	Guideline	Washington, DC, USA
3	Diagnosis and management of epilepsy in adults [24]	2015	SIGN	Guideline	Edinburgh, UK
4	Fertility: assessment and treatment for people with fertility problems [25]	2013	NICE	Guideline	London, UK
5	Guideline No. 410: Prevention, Screening, Diagnosis, and Pregnancy Management for Fetal Neural Tube Defects [26]	2021	SOGC	Guideline	Ottawa, ON, Canada
6	Guideline No. 427: Folic Acid and Multivitamin Supplementation for Prevention of Folic Acid Sensitive Congenital Anomalies [27]	2022	SOGC	Guideline	Ottawa, ON, Canada
7	Maternal and child nutrition [28]	2014	NICE	Guideline	London, UK
8	Pre-conception Folic Acid and Multivitamin Supplementation for the Primary and Secondary Prevention of Neural Tube Defects and Other Folic Acid-Sensitive Congenital Anomalies [29]	2015	SOGC	Guideline	Ottawa, ON, Canada
9	Guideline of preconception and prenatal care (2018) [30]	2018	Obstetrics Subgroup, Chinese Society of Obstetrics and Gynecology, Chinese Medical Association	Guideline	Beijing, China
10	AAFP Releases Position Paper on Preconception Care [31]	2016	AAFP	Guideline	Leawood, KS, USA
11	Preconception and prenatal folic acid supplementation [32]	2024	Laura M Goetzl et al.	Best Practice	Houston, TX, USA
12	Chinese Multidisciplinary Expert Consensus on Rational Clinical Folic Acid Supplementation [33]	2021	Chinese Multidisciplinary Expert Consensus Writing Group on Folic Acid	Expert Consensus	Baltimore, MD, China
13	Expert consensus on preconception care (2023) [34]	2023	Li, Li et al.	Expert Consensus	Beijing, China
14	Interconception Care for Primary Care Providers: Consensus Recommendations on Preconception and Postpartum Management of Reproductive-Age Patients with Medical Comorbidities [35]	2021	S. Michelle Ogunwole et al.	Expert Consensus	Baltimore, MD, USA
15	Prepregnancy Counseling [36]	2019	ACOG	Expert Consensus	Washington, DC, USA
16	Folic Acid Supplementation to Prevent Neural Tube Defects US Preventive Services Task Force Reaffirmation Recommendation Statement [37]	2023	US Preventive Services Task Force	Recommended Practice	Rockville, MD, USA
17	Good clinical practice advice: Micronutrients in the periconceptional period and pregnancy [38]	2019	FIGO	Recommended Practice	London, UK

Abbreviations: ACOG, American College of Obstetricians and Gynecologists; SIGN, Scottish Intercollegiate Guidelines Network; NICE, National Institute for Health and Care Excellence; SOGC, Society of Obstetricians and Gynaecologists of Canada; AAFP, American Academy of Family Physicians; FIGO, International Federation of Gynecology and Obstetrics.

**Table 2:** Results of quality evaluation of included guidelines (*n* = 10).

No.	Percentage of Standardized Domains (%)						Domains with >60% (Number)	Domains with <30% (Number)	ICC	Level
	Scope and Purpose	Stakeholder Involvement	Rigor	Clarity	Applicability	Independence				
1 [22]	69.44	66.67	30.21	94.44	37.50	8.33	3	2	0.908	B
2 [23]	72.22	33.33	45.83	86.11	39.58	8.33	2	1	0.880	B
3 [24]	80.56	75.00	76.04	91.67	95.83	100.00	6	0	0.879	A
4 [25]	88.89	83.33	94.79	100.00	95.83	100.00	6	0	0.773	A
5 [26]	94.44	66.67	56.25	94.44	54.17	83.33	4	0	0.973	B
6 [27]	94.44	69.44	55.21	94.44	54.17	83.33	4	0	0.775	B
7 [28]	88.89	86.11	96.88	100.00	95.83	100.00	6	0	0.815	A
8 [29]	94.44	66.67	56.25	94.44	54.17	83.33	4	0	0.832	B
9 [30]	80.56	61.11	10.42	66.67	27.08	8.33	3	3	0.798	C
10 [31]	58.33	25.00	0.00	69.44	35.42	8.33	1	3	0.845	C

Notes: Grade A, recommended; Grade B, recommended with modifications; Grade C, not recommended.

**Table 3:** Results of quality evaluation of expert consensus, best practices, and recommended practices (*n* = 7).

No.	Literature Type	Item 1 ^1^	Item 2 ^2^	Item 3 ^3^	Item 4 ^4^	Item 5 ^5^	Item 6 ^6^	Inclusion
11 [32]	Best Practice	Yes	Yes	Yes	Yes	Yes	No	Yes
12 [33]	Expert Consensus	Yes	Yes	Yes	Yes	Yes	No	Yes
13 [34]	Expert Consensus	Yes	Yes	Yes	Yes	Yes	No	Yes
14 [35]	Expert Consensus	Yes	Yes	Yes	Yes	Yes	No	Yes
15 [36]	Expert Consensus	Yes	Yes	Yes	Yes	Yes	No	Yes
16 [37]	Recommended Practice	Yes	Yes	Yes	Yes	Yes	No	Yes
17 [38]	Recommended Practice	Yes	Yes	Yes	Yes	Yes	No	Yes

^1^ Item 1: Is the source of the viewpoint clearly indicated? ^2^ Item 2: Does the viewpoint originate from influential experts in the field? ^3^ Item 3: Is the proposed viewpoint centered on the interests of the relevant population? ^4^ Item 4: Is the stated conclusion based on the results of the analysis? Is the expression of the viewpoint logical? ^5^ Item 5: Are other existing literature references consulted? ^6^ Item 6: Are there inconsistencies between the proposed viewpoint and previous literature?

**Table 4:** Definitions of risk groups for NTDs.

Risk Category	Definition
low risk	Women without any of the moderate- or high-risk factors listed below.
moderate risk	Women with following personal or comorbid conditions (1 to 5), or whose male partners have the following personal conditions (1 and 2): (1) Personal or family history of other folate-sensitive congenital malformations (limited to specific anomalies such as cardiac, limb, cleft palate, urinary tract, and congenital hydrocephalus); (2) Family history of NTDs in first- or second-degree relatives; (3) Pre-gestational diabetes (type 1 or 2) with associated fetal teratogenic risk. Measuring red blood cell folate levels may be part of preconception assessment to determine multivitamin and folic acid supplement dosing strategies (1.0 mg folic acid supplement when RBC folate < 906 ng/mL; 0.4 to 0.6 mg folic acid supplement when RBC folate > 906 ng/mL), in conjunction with a multivitamin; (4) Use of medications with antifolate and teratogenic effects. These include certain anticonvulsants (e.g., carbamazepine, valproic acid, phenytoin sodium, primidone, phenobarbital), metformin, methotrexate, sulfasalazine, trimethoprim (a component of co-trimoxazole), and cholestyramine; (5) Maternal gastrointestinal malabsorption secondary to specific medical or surgical conditions that are proven to cause reduced red blood cell folate levels (e.g., Crohn’s disease or active celiac disease, gastric bypass surgery, advanced liver disease, renal dialysis, excessive alcohol consumption).
high risk	Women with a personal history of NTDs or a previous NTD-affected pregnancy or their male partners who have a personal history of an NTD.

**Table 5:** Summary of the best evidence of folic acid supplementation for prevention of NTDs in women of childbearing age.

Category	Evidence Content	Evidence Level (Grade)
1. Risks of Neural Tube Defects and the Role of Folic Acid	1.1 Risks of Neural Tube Defects [22,37]	5
	1.2 The Role of Folic Acid in Preventing Neural Tube Defects [22,23,32]	1
2. Timing Window for Neural Tube Closure	2.1 Timing of Neural Tube Closure and Significance of Preconception Folic Acid Intake [22,23,26,32,37]	5
3. Timing and Dosage of Folic Acid Supplementation	3.1 Balanced Diet, Rational Use of Folic Acid Supplements, and Food Fortification Are Effective Means to Improve Folic Acid Nutritional Status [33]	5
	3.2 Women without high-risk factors who are planning pregnancy or may become pregnant should begin daily intake of folate-rich foods and supplements containing 0.4 mg of folic acid at least three months before anticipated conception. This should continue until the end of the first trimester. For the second and third trimesters and during lactation, the recommended supplemental folate dose is 0.4 mg/day [22,23,25,28,29,33,34,35,36,37,38].	1
	3.3 Women in the moderate-risk group should consume folate-rich foods and take a daily multivitamin supplement containing 1.0 mg of folic acid, starting at least three months before conception. This regimen should be continued until 12 weeks of pregnancy. For the second and third trimesters and during lactation, the recommended supplemental folic acid dose is 0.4 mg/day [22,29,33].	1
	3.4 High-risk groups should consume folate-rich foods and begin daily supplementation with 4.0 mg of folic acid at least one month before conception, continuing through the 12th week of pregnancy. Since 4 mg formulations are unavailable domestically but 5 mg formulations exist, daily supplementation with 5 mg of folic acid is also acceptable. From 12 weeks of pregnancy onwards, continue daily supplementation with a multivitamin containing 0.4 to 1.0 mg of folic acid throughout the entire pregnancy and for 4 to 6 weeks postpartum or until breastfeeding ceases [22,23,24,29,33,35,36].	1
	3.5 Personalized supplementation: In special circumstances, supplement dosage may be increased or preconception supplementation duration extended as appropriate [22,33].	3
	3.6 Do not obtain high-dose folic acid supplementation through multivitamin intake [32,40].	5
	3.7 The tolerable upper intake level for folic acid in adults is generally 1 mg. A 4 mg prophylactic dose for women at high risk of NTD pregnancies is typically considered non-toxic in the short term [32,41].	5
4. Relationship between dietary folate and folic acid tablets	4.1 Dietary folate sources include asparagus, spinach, broccoli, soybeans, citrus fruits, dried fruits, beef liver, leafy greens, legumes, avocados, eggs, dairy products, barley, tofu skin, dried bean curd sticks, walnuts, garlic sprouts, peanuts, rapeseed, fennel, red amaranth, chrysanthemum greens, chicken eggs, and duck eggs [22,32,33].	1
	4.2 Dietary intake alone is insufficient; folic acid supplementation is necessary [29,33].	2
5. Folate-Related Testing	5.1 Folic acid metabolism testing is generally not recommended [27,32].	1
	5.2 Women of childbearing age taking folic acid supplements do not require monitoring of folate levels [32].	3

Notes: In the JBI evidence grading system used here, level 1 represents the highest level of evidence and level 5 the lowest.

## Data Availability

Not applicable.

## Supplementary Materials

The following supporting information can be downloaded at: https://www.mdpi.com/article/10.3390/nu18040641/s1, Table S1: List of studies excluded after full-text review with reasons.

## Appendix A

### Evidence Summary Retrieval Strategy

**Table A1:** PubMed Search Strategy.

No.	Search Expression	Results
#1	“Folic Acid” [Mesh] OR “Prenatal Nutritional Physiological Phenomena” [Mesh] OR “Preconception Care” [Mesh]	48,400
#2	Folate [Title/Abstract] OR “folic acid” [Title/Abstract] OR “tetrahydrofolate” [Title/Abstract] OR “vitamin B9” [Title/Abstract] OR “THF” [Title/Abstract] OR “Pregnancy Nutrition” [Title/Abstract] OR “Preconception Care” [Title/Abstract]	67,036
#3	#1 OR #2	87,295
#4	“Neural Tube Defects” [Mesh]	31,855
#5	“neural tube defect*” [Title/Abstract] OR “congenital abnormality*” [Title/Abstract] OR anencephaly [Title/Abstract] OR encephalocele [Title/Abstract] OR “spina bifida” [Title/Abstract] OR meningocele [Title/Abstract] OR myelomeningocele [Title/Abstract]	33,847
#6	#4 OR #5	49,355
#7	(((((guidelines as topic [MeSH]) OR guideline [Publication Type]) OR consensus [MeSH]) OR consensus development conferences as topic [MeSH]) OR consensus development conference [Publication Type]) OR standard of care [MeSH]	255,271
#8	(((((((((guideline* [Title]) OR guidance* [Title]) OR recommendation* [Title]) OR consensus* [Title]) OR “best practice*” [Title]) OR statement* [Title]) OR standard* [Title]) OR “practice parameter*” [Title]) OR “position paper” [Title]) OR “position stand” [Title]	363,818
#9	#7 OR #8	505,414
#10	#3 AND #6 AND #9	163
#11	#10 Filters: from 2013–2025	54

#: Search step number; *: Truncation symbol used in search terms.

**Table A2:** Embase Search Strategy.

No.	Search Expression	Results
#1	(‘maternal nutrition’/exp OR ‘maternal nutrition’) AND [embase]/lim	15,034
#2	(‘prepregnancy care’/exp OR ‘prepregnancy care’) AND [embase]/lim	3,430
#3	(‘folic acid’/exp OR ‘folic acid’) AND [embase]/lim	84,215
#4	#1 OR #2 OR #3	100,271
#5	(folate:ab, ti OR ‘folic acid’: ab, ti OR ‘tetrahydrofolate’: ab, ti OR ‘vitamin b9’:ab, ti OR ‘thf’:ab, ti OR ‘pregnancy nutrition’: ab, ti OR ‘preconception care’: ab, ti) AND [embase]/lim	68,114
#6	#4 OR #5	118,093
#7	‘neural tube defect’/exp	46,554
#8	(‘neural tube defect*’:ab, ti OR ‘congenital abnormality*’:ab, ti OR anencephaly: ab, ti OR encephalocele: ab, ti OR ‘spina bifida’: ab, ti OR meningocele: ab, ti OR myelomeningocele: ab, ti) AND [embase]/lim	34,767
#9	#7 OR #8	60,082
#10	(‘guideline*’:ti OR ‘guidance*’:ti OR ‘recommendation*’:ti OR ‘consensus*’:ti OR ‘best practice*’:ti OR ‘statement*’:ti OR ‘standard*’:ti OR ‘practice parameter*’:ti OR ‘position paper’:ti OR ‘position stand’:ti) AND [embase]/lim	352,942
#11	(‘practice guideline’/exp OR ‘practice guideline’) AND [embase]/lim	713,415
#12	#10 OR #11	944,239
#13	#6 AND #9	5,754
#14	#12 AND #13	276
#15	#14 AND (2013:py OR 2014:py OR 2015:py OR 2016:py OR 2017:py OR 2018:py OR 2019:py OR 2020:py OR 2021:py OR 2022:py OR 2023:py OR 2024: py OR 2025:py)	124

#: Search step number; *: Truncation symbol used in search terms.

**Table A3:** CINAHL Search Strategy.

No.	Search Expression	Results
#1	MH “Prepregnancy Care” OR MH “Prenatal Nutritional Physiology” OR MH “Folic Acid”	10,646
#2	Title (folate OR folic acid OR tetrahydrofolate OR vitamin B9 OR THF OR Pregnancy Nutrition OR Preconception Care) OR Abstract (folate OR folic acid OR tetrahydrofolate OR vitamin B9 OR THF OR Pregnancy Nutrition OR Preconception Care)	12,508
#3	#1 OR #2	16,525
#4	MH “Neural Tube Defects”	2,938
#5	TI (neural tube defect OR congenital abnormalities OR anencephaly OR encephalocele OR spina bifida OR meningocele OR myelomeningocele) OR AB (neural tube defect OR congenital abnormalities OR anencephaly OR encephalocele OR spina bifida OR meningocele OR myelomeningocele)	7,496
#6	#4 OR #5	9,030
#7	MH “Practice Guidelines” OR MH “Consensus” OR PT practice guidelines	107,901
#8	TI (guideline* OR guidance OR recommendation* OR consensus* OR best practice* OR statement* OR standard* OR practice parameter* OR position paper OR position stand)	166,501
#9	#7 OR #8	231,224
#10	#3 OR #6	24,175
#11	#9 AND #10	659
#12	#9 AND #10 (Limiters—Publication Date: 1 January 2013–18 September 2025)	422

#: Search step number; *: Truncation symbol used in search terms.

**Table A4:** Web of Science Search Strategy.

No.	Search Expression	Results
#1	(TI = (“Folic Acid” OR folate OR “tetrahydrofolate” OR “vitamin B9” OR “THF”)) OR AB = (“Folic Acid” OR folate OR “tetrahydrofolate” OR “vitamin B9” OR “THF”)	128,589
#2	(TI = (“neural tube defect*“ OR “congenital abnormality*” OR anencephaly OR encephalocele OR “spina bifida” OR meningocele OR myelomeningocele)) OR AB = (“neural tube defect*” OR “congenital abnormality*” OR anencephaly OR encephalocele OR “spina bifida” OR meningocele OR myelomeningocele)	41,809
#3	TI = (guideline* OR guidance* OR recommendation* OR consensus* OR “best practice*” OR statement* OR standard* OR “practice parameter*” OR “position paper” OR “position stand”)	793,798
#4	#1 AND #2	4,436
#5	#3 AND #4	97
#6	#3 AND #4 and 2025 or 2024 or 2023 or 2022 or 2021 or 2019 or 2018 or 2017 or 2016 or 2015 or 2014 or 2013 (Publication Years)	36

#: Search step number; *: Truncation symbol used in search terms.

**Table A5:** CNKI Search Strategy.

No.	Search Expression	Results
#1	(Title: “Prenatal Nutrition” + “Prenatal Care” + “Folic Acid” + “Vitamin B9” (exact)) OR (Abstract: “Prenatal Nutrition” + “Prenatal Care” + “Folic Acid” + “Vitamin B9” (exact))	29,792
#2	(Title: “Birth Defects” + “Neural Tube Defects” + “Neural Tube Malformations” + “Neural Tube Defect Syndrome” (exact)) OR (Abstract: “Birth Defects” + “Neural Tube Defects” + “Neural Tube Malformations” + “Neural Tube Defect Syndrome” (exact))	15,784
#3	(Title: “Guidelines” + “Consensus” + “Evidence” + “Standards” + “Recommendations” + “Draft” + “Norms” + “Statements” (exact))	1,249,959
#4	#1 AND #2 AND #3	11

Note: Timeframe limited to 1 January 2013–18 September 2025.

**Table A6:** Wanfang Search Strategy.

No.	Search Expression	Results
#1	((Title or Keywords: (“Birth Defects” or “Neural Tube Defects” or “Neural Tube Malformations” or “Neural Tube Defect Syndrome”) or Abstract: (“ birth defects” or “neural tube defects” or “neural tube malformations” or “neural tube defect syndrome”)) AND (Title or Keywords: (“preconception nutrition” or “preconception care” or “folic acid” or “vitamin B9”) or Abstract: (“ Prenatal Nutrition” or “Prenatal Care” or “Folic Acid” or “Vitamin B9”)) AND (Title:(“Guidelines” or “Consensus” or “Evidence” or “Standards” or “Recommendations” or “Draft” + ”Specifications” or “Statements”))) and Publication Date: 2013–2025	18

**Table A7:** VIP Search Strategy.

No.	Search Expression	Results
#1	(M = (“birth defects” or “neural tube defects” or “neural tube malformations” or “neural tube defect syndrome”) OR R = (“birth defects” or “neural tube defects” or “neural tube malformations” or “neural tube defect syndrome”)) AND (M = (“Prenatal Nutrition” or “Prenatal Care” or “Folic Acid” or “Vitamin B9”) OR R = (“prenatal nutrition” or “prenatal care” or “folic acid” or “vitamin B9”)) AND (T = (“guidelines” or “consensus” or “evidence” or “standards” or “recommendations” or “draft” + ”specifications” or “statements”))	18

Note: Time period limited to 1 January 2013–18 September 2025.

**Table A8:** Search Strategy for Guidelines, Expert Consensus, Recommended Practices, and Best Practices.

Search Terms	Database	Results
“Folic Acid”/folate/”tetrahydrofolate”/”vitamin B9”/folacin/”Preconception Care”/”Pregnancy Nutrition”, “neural tube defect”/anencephaly/encephalocele/”spina bifida”/meningocele/myelomeningocele	GIN (Guidelines Network)	19
	NICE * (Guidelines Network)	61
	SIGN (Guidelines Network)	23
	CMA^1^ (Guidelines Network)	5
	NZGG ** (Guidelines Network)	2
	ACOG	23
	ACMG	2
	FIGO	7
	SOGC	13
	UpToDate	1
“Folic Acid”/”Vitamin B9”/”Preconception Care”/”Preconception Nutrition”/”Neural Tube Defects”/”Neural Tube Malformations”/”Anencephaly”	Medlive (Guidelines Network)	1

* Check “guidance” “NICE advice”. ** Take the results under “Guides and standards”. Notes: (1) GIN: Guidelines International Network; SIGN: Scottish Intercollegiate Guidelines Network; CMA^1^: Canadian Medical Association; NICE: National Institute for Health and Care Excellence; NZGG: New Zealand Guidelines Group; ACOG: American College of Obstetricians and Gynecologists; ACMG: American College of Medical Genetics and Genomics; FIGO: International Federation of Gynecology and Obstetrics; SOGC: Society of Obstetricians and Gynaecologists of Canada; (2) Additional databases searched with no results retrieved: Chinese Medical Ace Base; CMA^2^ (Chinese Medical Association); CNA (Chinese Nursing Association); AHRQ (Agency for Healthcare Research and Quality); NHMRC (National Health and Medical Research Council); QCG (Queensland Clinical Guidelines); ACPG (Australian Clinical Practice Guidelines); AAPF (American Academy of Family Physicians); AAP (American Academy of Pediatrics).
